# Sectoral networks and macroeconomic tail risks in an emerging economy

**DOI:** 10.1371/journal.pone.0190076

**Published:** 2018-01-02

**Authors:** Pedro P. Romero, Ricardo López, Carlos Jiménez

**Affiliations:** 1 Department of Economics, Universidad San Francisco de Quito, Quito, Pichincha, Ecuador; 2 Department of Mathematics, Universidad San Francisco de Quito, Quito, Pichincha, Ecuador; Universidad de Castilla-La Mancha, SPAIN

## Abstract

This paper aims to explain the macroeconomic volatility due to microeconomic shocks to one or several sectors, recognizing the non-symmetrical relation in the interaction among the Ecuadorian economic sectors. To grasp the economic structure of this emerging economy, a statistical analysis of network data is applied to the respective input-output matrix of Ecuador from 1975 until 2012. We find periods wherein the production of domestic inputs is concentrated in a few suppliers; for example, in 2010, the concentration significantly affects sectors and their downstream providers, thus influencing aggregate volatility. Compared to the US productive structure, this emerging economy presents fewer sectors and degree distributions with less extreme fat-tail behavior. In this simpler economy, we continue to find a link between microeconomic shocks and aggregate volatility. Two new theoretical propositions are introduced to formalize our results.

## Introduction

The impact of aggregate volatility, both economically and socially, influences the political and economic strategies of a country. Timely analysis and preparation to avoid the profound impacts of volatility are desired, but its estimation and calculation are not trivial and represent a major challenge. Aggregate volatility has been calculated in classical macroeconomics using the central limit theorem, which assumes that all economic sectors are equally important within an economy and which suggests that while the economy has more sectors, the effect of aggregate volatility is minimized. "This argument, however, ignores the presence of interconnections between different firms and sectors, functioning as a potential propagation mechanism of idiosyncratic shocks throughout the economy." [1]

Similarly, [2] shows that "Because of random growth at the micro level, the distribution of firm sizes is very fat-tailed… That fat-tailedness makes the central limit theorem break down, and idiosyncratic shocks to large firms… affect aggregate outcomes." This debate is at the core of the papers in [3] and [4,5].

More recently, [6], [7], [1], and [8] analyzed the effects of the interrelationships between the economic sectors and their effect on aggregate volatility in the U.S. In fact, [8] modeled tail comovements of the input-output linkages to support their argument that a reduction in aggregate volatility, such as the one experienced by the U.S. during the ‘Great Moderation,’ could occur in tandem with an increase in the likelihood of large economic downturns (see also [9–11]).

Conversely, [12] analyzed the industrial production index of the U.S. and extended the model of [13] by including capital in the production function in addition to the input-output variable (for an earlier dynamic model, see [14]). The main interest of that paper was to separate the common versus sectoral shock effects during the ‘Great Moderation’ period; the researchers found that the period was characterized by a reduction in the relevance of common shocks to aggregate volatility in favor of sectoral ones.

In this paper, we analyze the structure of production with a special focus on the sectoral linkages of the input-output matrix of an emerging country such as Ecuador. We found that emerging economies are less complex than more developed ones, such as the U.S.; when we compare the number of economic sectors, the exponents of the fat tails of the distribution of output degrees are less noticeable. In addition, since 2000, there has been an increase in the input-output network connectivity, thus increasing the level of criticality due to microeconomic shocks diffused through the network.

In Ecuador, there have been studies aimed at understanding the impact that a microeconomic shock would have; [15] analyzes the "Impact on the Ecuadorian economy by exogenous shocks on the agricultural sector (using the Input-Output method for the year 2007),” wherein they assert that “…the impact analysis is independent of the intersectoral analysis" [15]. In this study, we show that this assertion is false.

In [16], the breadth of products exported by Ecuador is shown, as are their interconnections. There is evidence that the major exports of Ecuador are produced by peripheral sectors but have certain interconnections with other sectors, suggesting that there are possibilities for the development of more economic sectors. The generalization of this approach to study economic development can be found in [17] and [18].

In [19], a study of how the relations between economic sectors can boost the economy through backward and forward linkages is presented, obtaining the demand and supply of the inputs of a sector, respectively. In this paper, we develop an additional step in analyzing the aggregate volatility, considering the interaction of economic sectors.

Empirical evidence for France at the sectoral and firm levels also shows that firm linkages contribute as much as three times more than direct shocks in aggregate fluctuations [20]. For other major countries in the OECD, how the international economic crisis of 2007–2009 was not exogenous but rather a consequence of the evolution of input-output structures from being dominated by manufacturing toward more service sectors, especially the financial sector, is presented in [21]. Finally, another related study that considers geographical links and distinguishes between supply- and demand-side shocks in the production network is [22].

This paper is organized as follows. In the next section, a model of an input-output economy is introduced. We then present the methodology of network graphs and how is applied to the input-output matrix; then, the results obtained, and the implications of the presence of heavy tails in the distributions of the data are analyzed. Subsequently, we introduce our two main propositions to formalize our results, and discuss the economic policy implications derived from them. Finally, we present the conclusions.

## Materials and methods

### A model of an input-output economy

In this section, we present a static model of an input-output neo-classical economy, which is a canonical version introduced by [13] and applied by [1,7,23].

Here, an input-output economy describes a market process wherein firms produce within different industry sectors. In their production processes, these firms demand inputs from other firms within or outside their industry sectors. Thus, there is intra-sectoral and inter-sectoral trade. In addition to inputs from other firms, a given firm also demands labor, and the output is subject to constant returns to scale and Harrod-neutral productivity exogenous shocks in this Cobb-Douglas production technology. Capital has not been included; otherwise, the required model must be dynamic.

**Fig 1:**
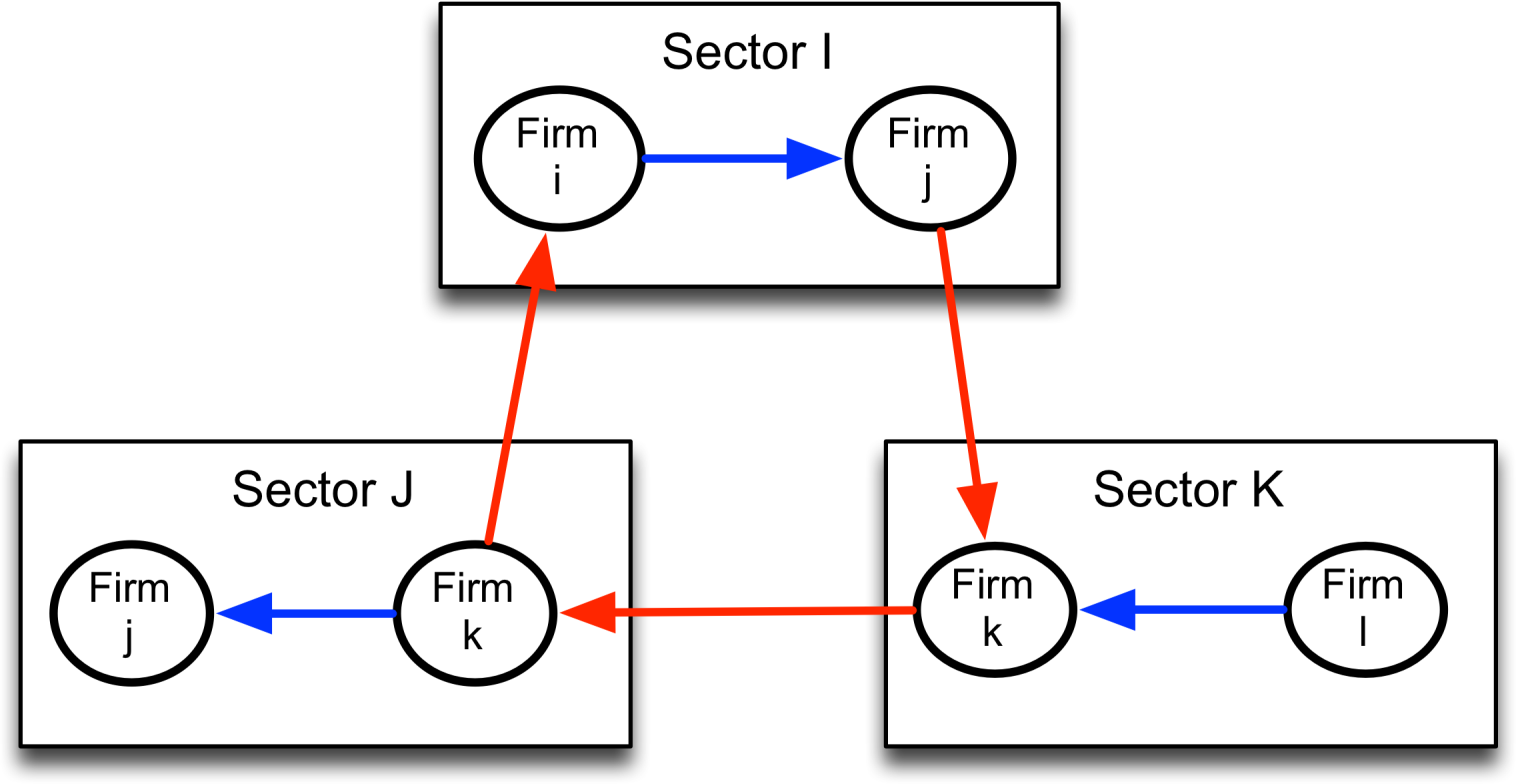
Network example. A stylized input-output network for firms and industries; blue arrows indicate intra-sectoral trade, and red arrows indicate inter-sectoral trade.

The input-output relationships within and outside industrial sectors form the network through which firms interact. This feature is key for our empirical specification, since it allows us to capture the heterogeneity embedded in the input-output matrices. Although the model is static, the different years available in our database let us study the change in the network of the input-output relationships of the Ecuadorian economy over a span of more than 30 years. We depict a basic version of this networked economy in Fig 1.

In this model, there are n industrial sectors represented by *I*_*n*_ = {1,2,…,*n*}. Each sector is subject to technology shocks *z*_*i*_ that are independent and identically distributed (i.i.d.) across sectors, and the distribution of log(*z*_*i*_) ≡ *φ*_*i*_ is represented by *F*_*i*_. By assumption, these shocks present a symmetric distribution around zero and have finite variance normalized to one. In addition, the input-output matrix *A*_*n*_ contains a typical element *a*_*ij*_, which represents the technical coefficients that relate the proportion of good j in the inputs of firms in sector i. Since there is intra-sectoral trade, we have *a*_*ii*_ > 0. Thus, the input-output network is a weighted directed graph, wherein an element of A represents the weights from sector j to sector i. The sectoral production behaves according to a Cobb-Douglas technology.

$$y_{i}=z_{i}^{\alpha }\;l_{i}^{\alpha }\prod_{j\epsilon {ℵ}_{i}}x_{ij}^{(1-\alpha )a_{ij}}$$ (1)

The participation of the labor factor, *l*_*i*_, in the output is *α ϵ* (0,1). Here, ℵ_*i*_ represents the j input providers of firms in sector i. The quantity of commodity j demanded by producers of commodity i is *x*_*ij*_. We assume constant returns to scale; thus, $\sum_{j=1}^{n}a_{ij}=1$.

In this economy, consumers or a representative household per sector has consumption preferences cover the goods of sector i, with a constant relative risk aversion (CRRA) utility but with *σ* = 1 and a linear disutility for labor.

$$u=\frac{1}{n}\sum_{i=1}^{n}(\ln c_{i}-\psi lnl_{i})$$ (2)

Given competitive markets, households maximize their utilities subject to a resource constraint; firms maximize profits subject to input prices and wages, and markets are cleared. In the S1 Appendix, we show that by following the foregoing process, the real value added or GDP in this economy is *y* = log(*GDP*) = *v*′*φ* and is expressed in vector form. The vector of influence *v* is:

$$v=\frac{\alpha }{n}{\left[I-(1-\alpha )A\prime \right]}^{-1}1$$ (3)

Thus, the Leontief inverse [*I* − (1 − *α*)*A*′]^−1^ captures the input-output network of weighted links between the different sectors and translates well to our empirical analysis of actual input-output matrices. We also have **1** as a vector with dimensions appropriate for the operation and *I* as the identity matrix. The real value added also includes the idiosyncratic shocks to sector productivity, which propagates to downstream sectors due to the sectoral network. If *α* is equal to one, then we have a simple economy with no input-output relationship and no shock transmission. When this case does not occur, some sectors are more connected than others, i.e., implying higher degrees; thus, these sectors are more prone than others to spread these shocks to the economy.

From the first-order conditions, we know that the input demand for the producer of good *j* is given by:

$$x_{ij}^{*}=\frac{(1-\alpha )a_{ij}p_{i}y_{i}}{p_{j}}$$ (4)

If we have two economies with different (real) income levels, A and B, where *Y*^*A*^ > *Y*^*B*^, then economy A will demand more than economy B from intermediate sectors. In fact, the derivative of input demands with respect to real output is positive. Hence, more developed economies will demand inputs from more sectors than a less developed economy.

Differences in capital intensity will also matter. Economies that exhibit distinct participation of inputs in national income will be asymmetrically affected by input-output relationships. In our case, variations in alpha in Eq 4 are negative or, in other words, they reduce input demands (see S1 Appendix).

### Data and definitions

To conduct this study, we use the input-output matrices presented by the Central Bank of Ecuador for the following years: 1975, 1980, 1985, 1990, 1993, 1995, 2007, 2010 and 2012.

The methodology used to build these matrices varies over time; prior to 1995, the matrices had 34 economic sectors; in 2007, the matrices expanded to 71 economic sectors; and in 2012, these 71 economic sectors were maintained, and an additional detailed matrix with 245 economic sectors is presented. These changes were due to an increase in the scope of the data by including more digits of the ISIC, initially from two to three digits, and then from three to four digits (see [24]). In the detailed 2012 matrix, the economic sectors that are most disaggregated are the following: Tubers, vegetables, melons and fruits (26 subsectors); Business services and production (17 subsectors); Transportation and storage services (11 subsectors); Live animals and animal products (11 subsectors); Oilseeds (10 subsectors); Machinery, equipment, and electrical appliances (8 subsectors); and Cereals (7 subsectors).

It is noteworthy that the Central Bank of Ecuador has no information on input-output matrices between 1996 and 2006. Between 1998 and 2000, the country was experiencing a financial crisis with harsh social consequences, which led to an emigration of both low-skilled and highly skilled workers; within the latter group, central bank personnel were included.

Additionally, to calculate the parameters of the heavy tail for the weighted degrees of production of the first and second order, we implemented the code developed by [25].

To measure *aggregate volatility*, we specify the criteria as follows: firstly, doing an analysis of heavy tails through maximum likelihood estimators and, secondly, by using the vector of influence introduced in the model section.

Let us begin with *first-order connections* that consider the direct relationship between one sector and all sectors with which it trades. The input-output matrix A comprises these connections. Below, we provide more precise definitions emphasizing economic relationships, since we are using graph-theoretical concepts. For instance, when we discuss the degree of demand, it is the same as the weighted indegree of a node or the element of the network; meanwhile, the degree of production is the same as the weighted outdegree of a node.

First-order weighted degree of demand: The degree of the input demand of sector *i* is the sum of the *j-th* columns of the matrix of technical coefficients of the input-output matrix A; it is denoted as $d_{i}^{c}$, where superscript *c* is identified as the degree of demand, and the subscript *i* represents the *i-th* sector:

$$d_{i}^{c}=\sum_{j=1}^{n}a_{ij}$$ (5)

For the subsequent analysis, we do not normalize $d_{i}^{c}$.

First-order weighted degree of production: The degree of production of a sector *j* is the sum of the *i-th* rows of the matrix of technical coefficients of the input-output matrix *A*, denoted as $d_{j}^{p}$, where the superscript *p* is identified as production, and the subscript *j* represents the *j-th* sector. To perform calculations of the degree of production, the *A*_*n*_ matrices are normalized to compare the distributions between different periods, thus standardizing the data. Once standardized, the following calculation is used:

$$d_{j}^{p}=\sum_{i=1}^{n}a_{ij}$$ (6)

Next, the estimation of the density distribution of the degrees of demand and production is performed through a Gaussian kernel, which is a function defined by:

w(t,h)=12πhe−(t22h2),−∞<t<∞, (7)

where *h* is the bandwidth or smoothing constant, and t is equal to *x* − *x*_*i*_, in which x is a random variable and *x*_*i*_ are the observations [26,27].

A quantity *x* obeys or follows a power law if the probability is given by the function p(x) ∝ *x*^−*β*^. The function noted diverges when *x* tends toward zero; therefore, it must be defined as a minimum value. We will show that, in our data, the degrees of production appear to follow a power law, for which the parameter is defined and the goodness of fit of the power law is measured.

The parameter of the heavy tail of first order degrees of production, through maximum likelihood estimators for the case of a continuous distribution, is defined as:

$$\hat{\beta }=1+n{\left[\sum_{i=1}^{n}ln\frac{x_{i}}{x_{min}}\right]}^{-1}$$ (8)

where *x*_*i*_, *i* = 1 … *n*, are the observed data for all *x* such that *x*_*i*_ ≥ *x*_*min*_. From now on, we refer to $\hat{\beta_{1}}$ as the parameter for connections of the first-order weighted outdegrees.

We cannot apply the same strategy as in [6,7] to compute the minimum value of *x* (*x*_*min*_) for the estimation of a heavy tail due to the small number of sectors present in the Ecuadorian input-output matrices; instead, we adopt the strategy proposed by [25] who estimate *x*_*min*_ by the following Kolmogorov-Smirnov (KS) statistic:

$$D=\max_{x\geq x_{\min}}\left|S(x)-P(x)\right|$$ (9)

where *S(x)* is the cumulative density function of the observed data greater than *x*_*min*_, and *P(x)* is the cumulative density function of the power law that best fits the empirical data in the region greater than *x*_*min*_. Hence, *x*_*min*_ minimizes D. In addition, *P(x)* is constructed using a resampling bootstrap, generating synthetic data that have similar distributions to the empirical data. To verify that the data follow a power law, we calculate the p-value to test the goodness of fit.

This p-value is obtained by measuring the fitness of the series to a power law, calculates the distance between the empirical data and the theoretical distribution, and uses the KS statistic. Based on the points made in [25], the p-value is estimated, first by assuming that for n observations, the observed data have *n*_*tail*_ observations with *x* at least greater than *x*_*min*_. Then, with probability *n*_*tail*_/*n*, a random number *x*_*i*_ is generated from a power law that has parameter $\hat{\beta }$ and a quantity of data equal to *n*_*tail*_. With probability 1-*n*_*tail*_/*n*, we uniformly select at random an element of the observed data with *x* < *x*_*min*_ to set *x*_*i*_ to that number. This process is repeated for all *i* = 1… *n*. The synthetic data follow a power law above *x*_*min*_ but a non-power law distribution below, as is the case of the observed data.

This semi-parametric method builds 1000 sets of theoretical data that have the same distribution as the empirical data and that allow one to compare them, obtaining a p-value that is the fraction denoting the times that the KS statistic for the theoretical data is greater than that for the empirical data. The criteria remain the same as those used by [25], who establish that a series follows a power law if the p-value is greater than 0.1.

We also need to specify concepts to analyze *second-order connections* in the network. These connections represent the indirect relationships that exist between sectors *k* and *i*, when sector *j* is connected to sectors *k* and *i*, but the former is not linked to i. For example, the iron sector trades directly with the construction sector, which, in turn, trades with the sector of real-estate services. Thus, there is only an indirect connection between the iron and real-estate sectors. These connections allow us to study what occurs when the price of iron increases. This behavior may reduce the amount of goods produced by the construction sector, which, in turn, affects the real-estate industry; this is the cascade effect that the second-order connections seek to measure.

Next, we define the *second-order weighted degree of production* of the *i-th* sector as the linear combination of the first-order weighted degrees of production by the matrix of technical coefficients:

$$q_{i}^{p}\equiv \sum_{j=1}^{n}d_{j}^{p}a_{ji}$$ (10)

where $d_{j}^{p}$ is the first-order weighted degree of production of the *j-th* sector, and *a*_*ij*_ is the component of the matrix of technical coefficients in the *j-th* location.

Finally, we estimate the parameter of the heavy tail for the second-order degrees of production using maximum likelihood estimators; this parameter is denoted by $\hat{\beta_{2}}$, again considering the process suggested in [25].

## Results and discussion

During our empirical analysis we will be comparing the results obtained for Ecuador with those better known of the United States. First of all, both the theoretical and the empirical model are adequate to study both type of economies. At least, formally the differences between the two countries could be interpreted as different positions in a wide range parameter space. Moreover, we believe such comparison makes our argument clearer for audiences not very familiar with developing and unknown countries like Ecuador.

### Analysis of input demand

In our analysis on the interconnections between sectors of the Ecuadorian economy by the demand for inputs, we applied a nonparametric estimation of the empirical density for degrees together with a smoothing Gaussian kernel.

**Fig 2:**
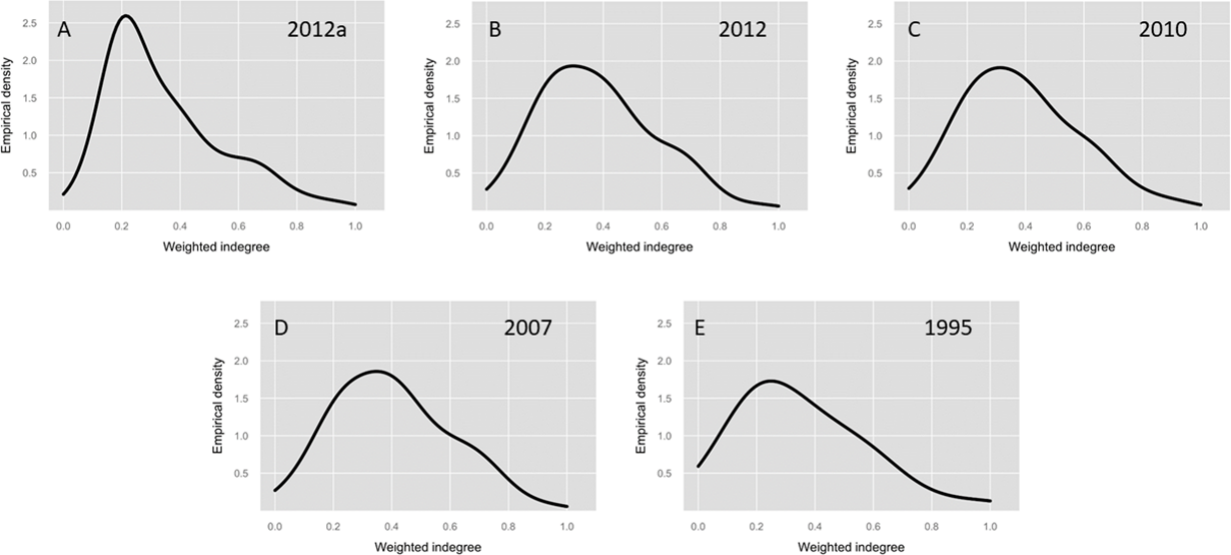
Density first-order weighted indegree input-output matrices 1995–2012. (A) Detailed matrix 2012, 245 economic sectors. (B) Summary matrix 2012, 71 economic sectors. (C) Matrix 2010, 71 economic sectors. (D) Matrix 2007, 71 economic sectors. (E) Matrix 1995, 34 economic sectors.

**Fig 3:**
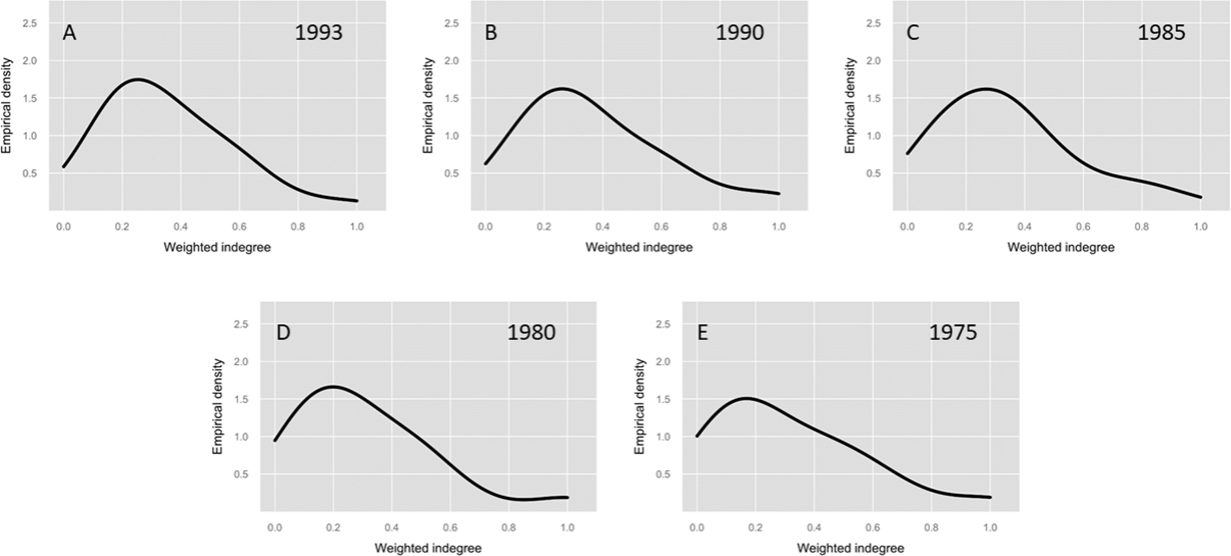
Density first-order weighted indegree input-output matrices 1975–1993. (A) Matrix 1993, 34 economic sectors. (B) Matrix 1990, 34 economic sectors. (C) Matrix 1985, 34 economic sectors. (D) Matrix 1980, 34 economic sectors. (E) Matrix 1975, 34 economic sectors.

Figs 2 and 3 plot the concentration of the empirical density for degrees of demand in all available years. The curves look similar in most periods, except for the detailed level matrix of 2012 (2012a) and those for 1995, 1980 and 1975, where the medians are lower than other periods, as shown in Table 1.

**Table 1:** Average, standard deviation, and median of the degrees of demand.

*Period*	*Number of Sectors*	*Average*	*Standard Deviation*	*Median*
*2012a*	245	0.3474	0.1983	0.2856
*2012*	71	0.3802	0.1883	0.3748
*2010*	71	0.3858	0.1923	0.3636
*2007*	71	0.4015	0.1928	0.3816
*1995*	34	0.4153	0.212	0.339
*1993*	34	0.3836	0.2111	0.3352
*1990*	34	0.3827	0.2397	0.3335
*1985*	34	0.3498	0.237	0.3337
*1980*	34	0.3644	0.2415	0.2771
*1975*	34	0.3763	0.2566	0.2976

These results imply that firms in different Ecuadorian economic sectors have historically imported, on average, between 60% and 75% of the inputs required for production. Additionally, when we analyze the concentration, we obtain that, on average, 66% of the sectors are within one standard deviation of the mean input share, while 91% of the sectors are within two standard deviations of the mean input share.

In the next section, we analyze the concentration that exists with respect to sectors that produce inputs for the remainder of the Ecuadorian sectors. There is a large difference in certain periods, where the concentration is much higher than that found in the first-order degrees of demand.

### Analysis of input supply

#### Degree of first-order production

**Fig 4:**
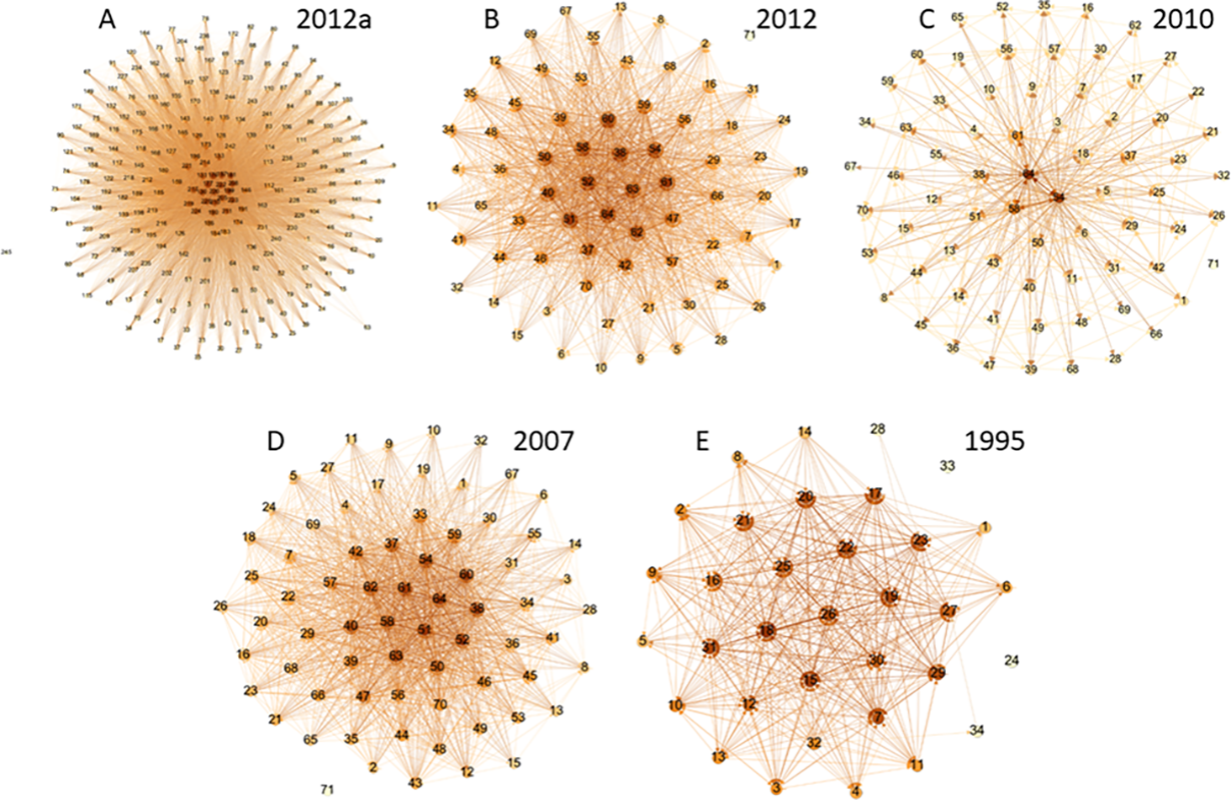
Network evolution of the input-output Ecuadorian matrix. (A) Detailed matrix 2012, 245 economic sectors. (B) Summary matrix 2012, 71 economic sectors. (C) Matrix 2010, 71 economic sectors. (D) Matrix 2007, 71 economic sectors. (E) Matrix 1995, 34 economic sectors. Each node represents a sector of the economy, and an edge has been mapped between two nodes when they have a percentage of trade equal to or greater than 1%.

Fig 4 shows the interconnections between the Ecuadorian economic sectors for the detailed level input-output 2012 data and summary input-output data for 2012, 2010, 2007 and 1995.

**Fig 5:**
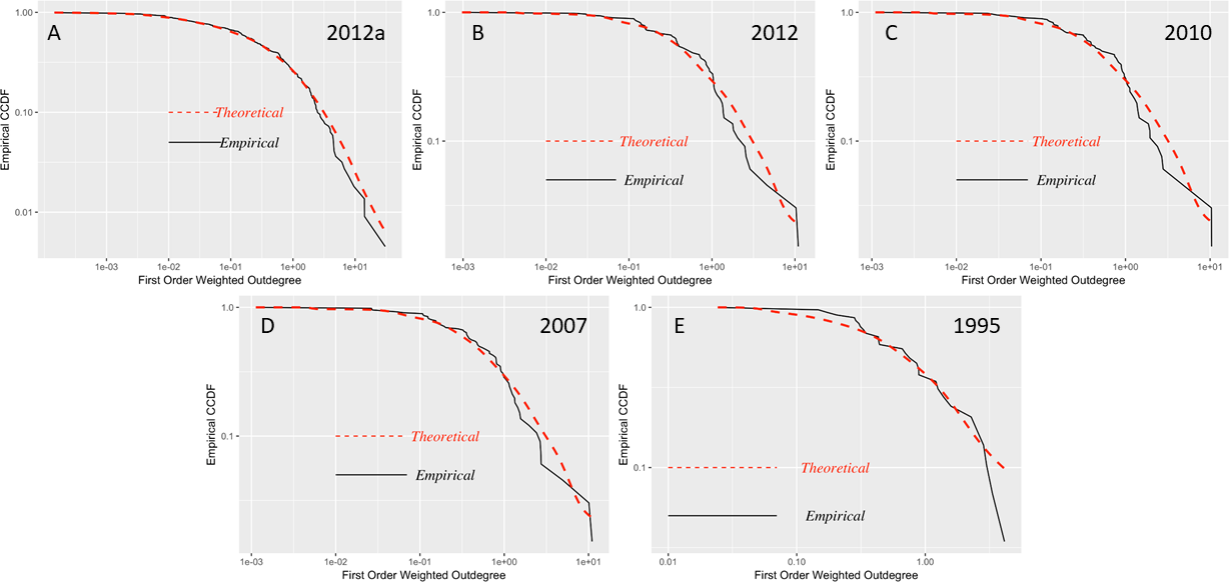
CCDF first-order weighted outdegree input-output matrices 1995–2012. (A) Detailed matrix 2012, 245 economic sectors. (B) Summary matrix 2012, 71 economic sectors. (C) Matrix 2010, 71 economic sectors. (D) Matrix 2007, 71 economic sectors. (E) Matrix 1995, 34 economic sectors. Log-log plots of the empirical counter-cumulative density functions (CCDF) of the weighted first-order outdegree.

**Fig 6:**
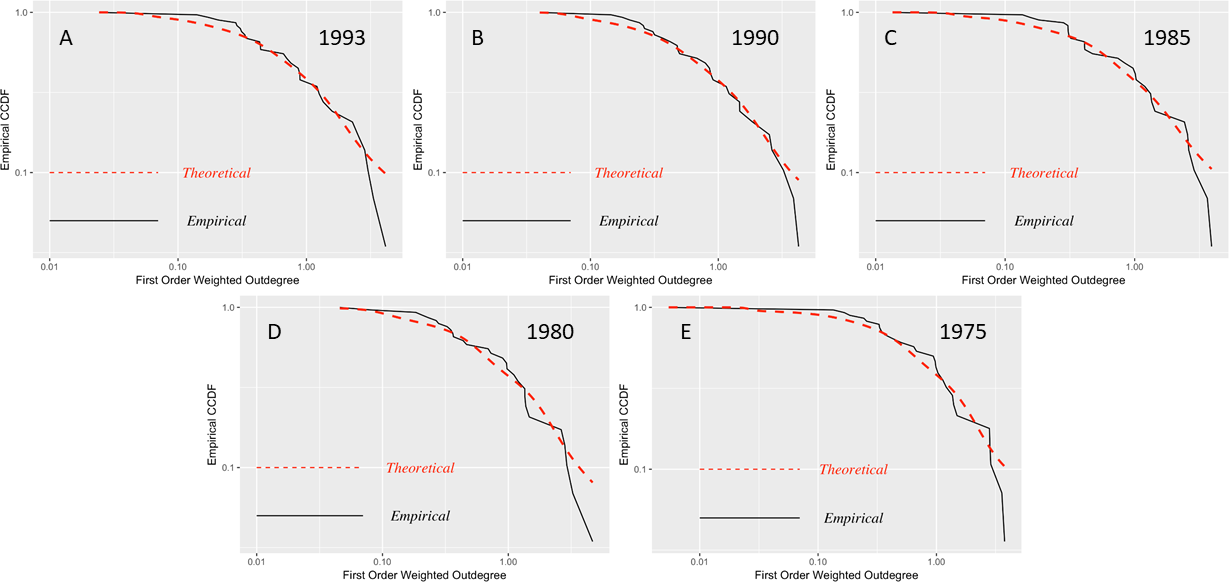
CCDF first-order weighted outdegree input-output matrices 1975–1993. (A) Matrix 1993, 34 economic sectors. (B) Matrix 1990, 34 economic sectors. (C) Matrix 1985, 34 economic sectors. (D) Matrix 1980, 34 economic sectors. (E) Matrix 1975, 34 economic sectors. Log-log plots of the empirical counter-cumulative density functions (CCDF) of the weighted first-order outdegree.

Examining Table 2 in chronological order, we observe that the quantity of sectors with a higher concentration of degrees of first-order production is decreasing, depending on the disaggregation of economic sectors. From 1980 to 1995, approximately 40% of the economic sectors have a higher concentration; between 2007 and 2012 (summary), there is an approximate concentration between 33% and 37%, respectively. However, for the detailed 2012 data, the concentration is only in 15.92% of sectors. To deepen our knowledge on the sectors that are becoming most connected, in Figs 5 and 6, we present the log-log plots of the empirical and theoretical distribution of density for the first-order production degrees.

**Table 2:** Power-law test for first-order degrees of production.

*Period*	*x*_*min*_	*Sectors*	*Sectors>x*_*min*_	${\hat{\beta }}_{1}$	*p-value*	*Standard error*
*2012a*	1.698	245	39	2.4215	0.453	0.0364
*2012*	0.8463	71	26	2.5977	0.768	0.0614
*2010*	0.8912	71	24	2.5934	0.95	0.0664
*2007*	0.7369	71	27	2.3721	0.97	0.0508
*1995*	0.6621	34	15	2.243	0.09	0.0829
*1993*	0.6622	34	15	2.2436	0.104	0.0829
*1990*	0.6805	34	14	2.2132	0.252	0.0867
*1985*	0.7421	34	14	2.2874	0.037	0.092
*1980*	0.6914	34	15	2.2865	0.071	0.0858
*1975*	0.1664	34	25	1.6385	0	0.0255

In Figs 5 and 6, we observe that the density distribution of the 2012 detailed matrix (2012a) compared with that for the same year but more aggregated (71 economic sectors) shows a greater difference from the theoretical distribution. This behavior is similar to that found by [1] in the U.S., where with a greater disaggregation of the input-output matrix, one can detect the increase in the interconnections between sectors and an impact of a microeconomic shock of at least double in size compared to that which can be obtained in more aggregated matrices.

Due to the behavior presented in Figs 5 and 6, we proceed to examine whether the concentration of first-order degrees of production have a heavy tail by applying maximum likelihood estimators as described in section 3.1. In Table 2, we present the results of the estimation of heavy tails for Ecuadorian input-output matrices.

The p-value obtained indicates that in only 1975, 1980 and 1985, the data does not follow a power law. These years include the first oil boom and its subsequent decline until the mid-1980s. Meanwhile, in the other periods, there is the presence of power-law behavior, particularly in 2007, 2010 and 2012. Previously, this behavior only occurred in 1990. Any difference between the summary and the detailed 2012 data, which are represented by a reduction in the exponent, may be attributed to the disaggregation of economic sectors. We should keep in mind that the results for 1975–1995 are based on two-digit ISIC sectorial classification, while data from 2007 to 2012 are based on three-digit ISIC to understand the limits of our comparative analysis.

The distribution of the first-order degrees of production is due to various factors that affected the Ecuadorian economy after the oil boom of the 1970s. In 1990, certain sectors related to manufacturing began to become the focus in national production, but over time, the diversification and expansion of these leading sectors have been reduced, while service sectors have become more prominent since the 2000s. Hence, in the detailed matrix for 2012, the 39 sectors that presented a power law are primarily service sectors, the top five more concentrated of which are the following sectors: trade services, employment services, road freight transportation services, services related to agriculture, and financial intermediation services of other depository institutions and SIFMI (Financial Intermediation Services Indirectly Measured). In 2010 and 2007, of the 24 and 26 sectors, respectively, that presented a power law, the three with the highest concentration were trade services, business services and production, and transport and storage services.

Conversely, in 1995, of the 15 sectors that presented a greater concentration, the five sectors with the highest concentration were basic metallic and nonmetallic mineral products; chemicals, plastics and rubber products; businesses services; machinery, equipment and transport equipment; and oil refining products.

There is a difference between the productive matrix arrays of 1995 and the matrices for 2007, 2010 and 2012; the latter present concentrations primarily in service sectors, while for 1995, the sectors with the highest concentration were manufacturing sectors. This finding is related to the data results of [21] from the UK, Japan, Germany, and France.

Despite the concentrations presented above in degrees of first-order production, it is important for this study to understand the volatility generated by the spreading of a microeconomic shock through indirect relationships between economic sectors. Next, we present the results of second-order production degrees.

#### Second-order degrees of production

In this analysis, we will discern how microeconomic shocks spread through the input-output network in the presence of any power-law distribution in these more indirect relationships between economic sectors.

**Fig 7:**
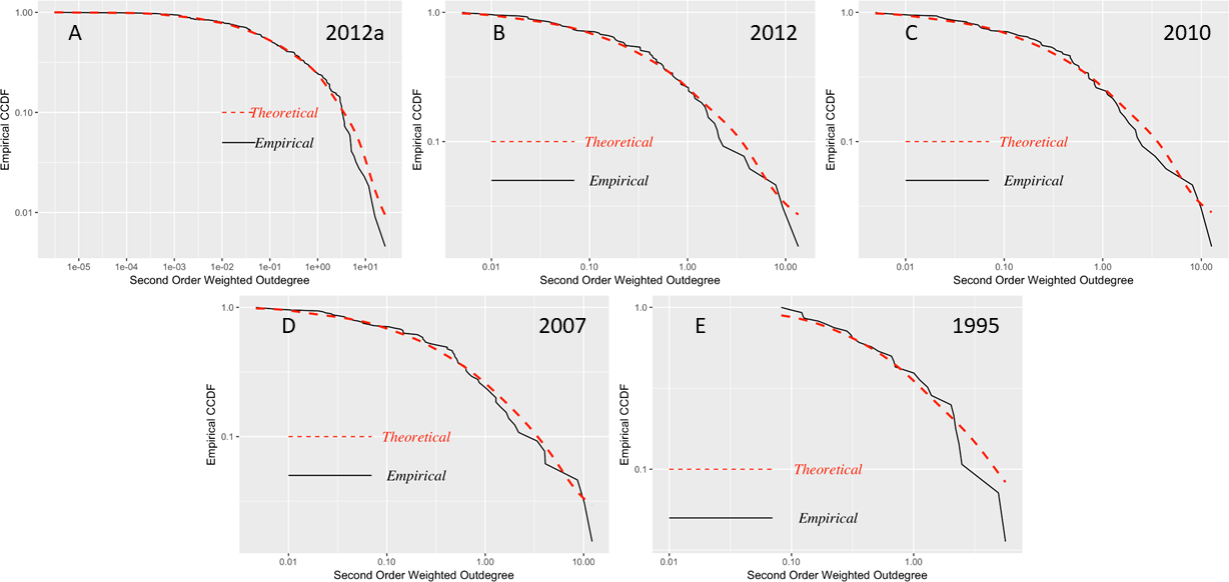
CCDF second-order weighted outdegree input-output matrices 1995–2012. (A) Detailed matrix 2012, 245 economic sectors. (B) Summary matrix 2012, 71 economic sectors. (C) Matrix 2010, 71 economic sectors. (D) Matrix 2007, 71 economic sectors. (E) Matrix 1995, 34 economic sectors. Log-log plots of the empirical counter-cumulative density functions of the weighted second-order outdegree.

**Fig 8:**
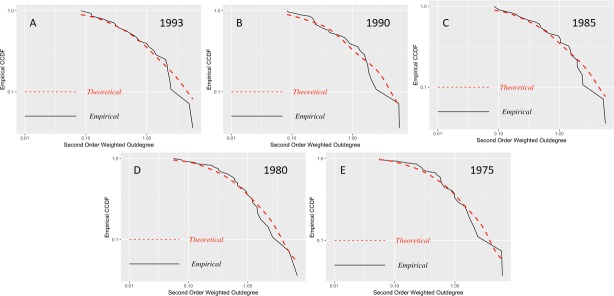
CCDF second-order weighted outdegree input-output matrices 1975–1993. (A) Matrix 1993, 34 economic sectors. (B) Matrix 1990, 34 economic sectors. (C) Matrix 1985, 34 economic sectors. (D) Matrix 1980, 34 economic sectors. (E) Matrix 1975, 34 economic sectors. Log-log plots of the empirical counter-cumulative density functions of the weighted second-order outdegree.

As can be observed in Figs 7 and 8, the second-order weighted degrees of production present distributions that differ from the theoretical densities, in particular, for 2007 and 2012. This finding may indicate that there are sectors with a high concentration of second-order connections, which can generate significant aggregate volatility in the event of a microeconomic shock. Then, we conducted the test to determine whether the distributions of second-order degrees of production follow a power law for each of the years in our sample, and we obtained the results shown in Table 3.

**Table 3:** Power law test for second-order degrees of production.

*Period*	*x*_*min*_	*Sectors*	*Sectors>x*_*min*_	${\hat{\beta }}_{2}$	*p-value*	*Standard error*
*2012a*	3.3164	245	20	2.7426	0.678	0.0871
*2012*	1.0199	71	16	2.1892	0.749	0.0743
*2010*	0.4668	71	29	1.9732	0.503	0.0336
*2007*	0.411	71	31	1.9441	0.894	0.0326
*1995*	0.1215	34	25	1.5851	0.004	0.0234
*1993*	0.1971	34	21	1.6593	0.021	0.0314
*1990*	0.1338	34	24	1.5975	0.003	0.0249
*1985*	0.0854	34	27	1.5221	0.001	0.0193
*1980*	0.5958	34	15	2.22	0.37	0.0813
*1975*	0.4766	34	17	2.13	0.507	0.0665

We can confirm that from 2007 to 2012 (summary and detailed), the distribution of the second-order outdegree follows a power law, as it does for 1975 and 1980. These last two years are not in agreement with what we found in the previous analysis. Their magnitude is higher than those for 2007 and 2010. A parsimonious explanation is that in the second half of 1970s, each economic sector may have had fewer direct providers but, due to the overall small number of indirect relationships of sectors, these providers were unavoidable and significant. This finding may also point to the economic dynamism generated by the oil boom, which increased second-order interconnections between different sectors. In contrast, between 1985 and 1995, the subsequent disappearance of this effect due in part to the ‘lost decade’ and the slow recovery in the early 1990s occurred. We do not have a similar explanation for 2007, 2010 and 2012; in contrast to 1980 and 1975, these periods show concentrations of first-order connections, which show that recent periods are more sensitive and that the aggregate volatility could be caused by microeconomic shocks spread through the input-output network, in addition to common shocks to the economy.

Given the foregoing results and to obtain a better grasp of the impact of aggregate volatility, in Table 4, we present a comparison between the calculations of the norm two of the vector of influence (‖***v***‖_2_) and the estimate of aggregate volatility defined in classical macroeconomic theory (1n) with n as the number of sectors. Two different values of *α* are evaluated in Eq 3 for the vector of influence, the first value making reference to the proportion typically used in the context of a developed economy, *α*_1_ = 0.3333 [1], and the second value corresponds to the proportion calculated for Ecuador, *α*_2_ = 0.57 (29). Moreover, the Euclidean norm of the vector of influence helps to determine the speed of the spreading of a shock in the event of a network, which represents the non-symmetric interaction between economic sectors.

**Table 4:** Analysis of the impact of aggregate volatility.

Period	1/(n)^(1/2)	||v1||2	||v1||2/(1/(n)^(1/2))	||v2||2	||v2||2/(1/(n)^(1/2))
2012a	0.0639	0.123	1.9247	0.0937	1.4664
2012	0.1187	0.2028	1.7084	0.1546	1.3024
2010	0.1187	0.2008	1.6916	0.1536	1.294
2007	0.1187	0.2009	1.6916	0.1543	1.2999
1995	0.1715	0.2201	1.2833	0.1882	1.0974
1993	0.1715	0.2204	1.2852	0.1883	1.0979
1990	0.1715	0.2256	1.3153	0.1904	1.1102
1985	0.1715	0.2196	1.2804	0.1882	1.0974
1980	0.1715	0.2292	1.3364	0.1907	1.1119
1975	0.1715	0.223	1.3004	0.1872	1.0915

We have computed two vectors of influence, ***v***_**1**_ and ***v***_**2**_; the results show that there are important differences between the estimate of aggregate volatility under the classical macroeconomic methodology and that using the vector of influence. The fact that the vector of influence based on the smaller alpha is higher than that originating from the Ecuadorian economy implies that, overall, this economy is not as volatile as the U.S. economy to the transmission of microeconomic shocks through the input-output network. Indeed, as [28] noted, countries that have grown faster between 1960 and 2000 have also experienced a greater incidence of financial crises compared to more stable economies.

If the rate of decay of volatility was behaving according to conventional macroeconomics, then the ratios in the fourth and sixth columns should be one; instead, we obtain ratios greater than 1 for both vectors of influence. In addition, we observe that between 1975 and 1995, the average ratio is 1.1, while this ratio is 1.3 for the 2007, 2010 and 2012 summary matrices in the last column. For 2012, we could also compute the ratio between the detailed and summary matrices, which is close to 0.61 for both vectors of influence. This result is higher than the ratio between $\left(1/\sqrt{245}\right)/(1/\sqrt{71})$, which is 0.54. This last finding implies that network effects are more relevant at higher levels of disaggregation, since conventionally, there should be a decline in the role of sectoral shocks for aggregate volatility of 46%; however, we only obtain a reduction of 7% in the data. Finally, the impact using the parameter of the U.S.(one third) is nearly double the estimate by traditional macroeconomics; using the Ecuadorian parameter, the impact is approximately an additional 47%. This finding reflects that this emerging economy presents a simpler economy than that which characterizes the more developed U.S. economy.

### The extent of the market and complexity

In our analysis thus far, we have shown that there are several economic characteristics that make a developing economy, such as Ecuador, different from a developed country such as the U.S. ([31] and see also [32] for an analysis of 155 countries). Firstly, in 2002, the number of economic sectors with a similar 4-digit ISIC code classification for the U.S. was 417 sectors, while Ecuador only had 245. Secondly, the relative contribution of labor is strikingly different. Thirdly, our analysis of power laws yields that this item has recently become a characteristic of the Ecuadorian economy in the 2000s but has been present in the U.S. for a while; for instance, [1] showed this characteristic with input-output data since 1972. We focus on the detailed matrix of 2012 and compare the shape parameter for first-order degrees, 2.42, with that for second-order degrees of 2.74; this increment reflects that there is not much decay in the rate of shock transmission in a developing economy. Thus, fewer economic sectors engender less dynamism to the benefit of greater stability.

Let us revise the formula for Eq 8 for the heavy-tail exponent, remembering that the formula is the same for both first- and second-order weighted outdegrees $\hat{\beta_{1}}$ and $\hat{\beta_{2}}$, where the random variable is the degrees in each case, *d*^*p*^ and *q*^*p*^. An economy with a smaller number of sectors, everything else being constant, should tend to yield lower estimates for the heavy tail. Whenever this assertion is not the case, the difference between heavier tails in the distribution of weighted outdegrees between economies with different levels of development should lie in a greater number of economic sectors with higher degrees or connectivity.

**Proposition 1** Two economies, A and B, will present different estimates for the heavy tail of the distribution of their outdegrees; in particular, one economy will have a lower estimate if it is more connected:

$$\sum_{i}^{n}ln\frac{d_{i}}{d_{min}}\;in\;A>\sum_{i}^{n}ln\frac{d_{i}}{d_{min}}\;in\;B.$$

Proof: To make our proposition more precise, now assume that two economies, A and B, have the same number, n, of economic sectors at a given level of disaggregation and that both present a similar minimum degree, that is, *d*_*min*_ is equal for A and B. Re-expressing either side of the above inequality as $\sum_{i}^{n}lnd_{i}-n*lnd_{min}$, the inequality simplifies to:

$$\sum_{i}^{n}lnd_{i}\;in\;A>\sum_{i}^{n}lnd_{i}\;in\;B$$

This equation implies that the magnitude of the elements of the sum to the left of the inequality is higher than for those to the right. Everything else being equal, there must be at least one sector with a higher degree in economy A than in economy B for the inequality to hold. Anything above this threshold will strengthen that relationship. **Q.E.D.**

In sum, for an economy to have a lower or more critical value for the exponent of the tail of the degree distribution, it is necessary for the economy to have more connections.

**Proposition 2** Economies with many economic sectors will have a lower value of $\hat{\beta }$ as long as they present more sectors with higher connectivity, that is, higher degrees.

Proof: Suppose that economy A has twice as many economic sectors than economy B, n_A_ = 2n_B_. Moreover, both economies present an economic sector with a similar minimum degree. Then, for economy A, it must be true that:

$$\sum_{i}^{2n_{B}}lnd_{i}>\sum_{i}^{n_{B}}lnd_{i}+n_{B}lnd_{min}$$

That is, the larger economy must have economic sectors that are more connected to others. An economy with more economic sectors has a higher threshold regarding its connectivity than when both economies are of the same size.

For example, let the degree sequence for economy A be {122223}, while for economy B, let the degree sequence be {112}; that is, *n*_*A*_ is 6, and *n*_*B*_ is 3. Given the degree sequences and that the minimum degree for A and B is 1, the result will be 3.871 > 0.693, as we would have expected. **Q.E.D**

What we have discussed thus far notes the relevance of link creation in this context. This process implies better opportunities for profit-seeking economic activities with local and global effects throughout the network [29–31].

The economic policy implications derived from our work are several. Firstly, to increase connectivity among sectors those frictions that negatively affect the costs in the labor and the inputs markets must be alleviated. For example, in Ecuador during the period being analyzed part-time labor contracts have been mostly banned by the legislation, and that has forced a lot of workers; between 40 and 50 percent of the labor force; to remain self-employed in the informal sector in very low value-added economic activities. Also, an autarkic perspective has characterized Ecuador’s foreign trade policy with tariffs still being an important tool for fiscal policy, which has increased the costs to import technology and other intermediate goods.

Moreover, an emerging economy like the one studied here is not as dynamic as one more developed, this is reflected in the fewer economic sectors present in the production structure. This implies that the extent of the market is more limited for the former economy than the latter. According to the World Intellectual Property Organization between the years 2000 and 2015, the maximum number of patents in a given year for Ecuador was 27 in 2003, while for the U.S. that number was a bit more than a half million in 2015. This is a ratio of almost twenty thousand times. Hence, to stimulate the flourishing of new economic sectors, more innovation is required in Ecuador. At this moment in Ecuador is more relevant to implement policies that eliminate obstacles to entrepreneurship to unleash innovation. For example, to reduce the time it takes to legally open a new business.

Finally, we emphasize that the main problem in Ecuador is not too much economic (or even financial) connectivity causing more volatility in the economy, instead, is the lack of connectivity causing an economy not growing at enough high rates to overcome poverty traps. What we mean by this is that Ecuador, also, lacks the institutional framework that helps entrepreneurs and investors to be more willing to take risks and start businesses than in a developed economy. Therefore, one important necessary reform is to establish an independent judicial system to guarantee rights and solve trials in an impartial manner. This will, also, insert the required efficiency to trade in the market.

## Conclusions

To obtain deeper understanding regarding why some countries are richer than others, it may be useful to compare modern economies at different stages of development. Input-output models could help us to uncover mechanisms that hamper the convergence of lagged economies toward more developed economies. Here, we studied the evolution of an emerging economy that contrasts with what is currently known of more sophisticated economies, which are usually the focus of research.

In this regard, our analysis describes how an emerging economy, in this case, Ecuador, has been changing its production structure in different stages. Ecuador is characterized as an economy that continues to need to import much of the inputs it needs for production, which makes it vulnerable to conditions in the international market. The concentration of the degrees of demand is not as high as those of production, such as is the case for the U.S. The degrees of production show higher concentrations over time and even more in the disaggregated matrix for 2012.

Ecuador’s input-output network has 245 sectors at 4-digits ISIC disaggregation, while that of the U.S. has 417 at the same level. In addition, our estimations for the tail exponents are larger than those found in the U.S., reflecting productive structures with different levels of criticality. However, we emphasize that our results note that the Ecuadorian economy is currently more sensitive when confronted with a microeconomic shock than it was before 2000, and, therefore, its impact would be more serious than the economic crisis that Ecuador experienced in 1999 [32].

## Supporting information

S1 Appendix Mathematical proofs. (DOCX)

S1 File IO matrices 1975–1995. (CSV)
