# Supplementary material for: Sectoral networks and macroeconomic tail risks in an emerging economy
Source: PLoS One. 2018 Jan 2;13(1):e0190076. doi: 10.1371/journal.pone.0190076 (PMC5749774; doi:10.1371/journal.pone.0190076)
Supplement: S1 Appendix — (DOCX) [file pone.0190076.s001.docx]

**S1 Appendix**

**Mathematical proofs**

Here we derive the equilibrium of the model.

Assumption: $\sum_{j=1}^{n} a_{ij}=1 \forall i$

$$I_{n}=\left\{ 1,2,\ldots,n \right\} sectors of the economy$$

$$Representative firm maximizes profits:$$

$$\max_{l_{i},y_{i},\left\{ x_{ij} \right\}\in I_{n}} p_{i}y_{i}-{hl}_{i}-\sum_{j=1}^{n} p_{j}x_{ij}$$

$subject to: y_{i}=z_{i}^{\alpha}l_{i}^{\alpha}\prod_{j\in\aleph_{i}} x_{ij}^{\left( 1-\alpha\right)a_{ij}} \alpha\in\left( 0,1 \right)$

$$\aleph_{i}:set of input providers to firm i$$

$$h:market wage$$

$$p_{i}:price of good produce by sector i$$

$$p_{i}z_{i}^{\alpha}l_{i}^{\alpha}\prod_{j\in\aleph_{i}} x_{ij}^{\left( 1-\alpha\right)a_{ij}}-{hl}_{i}-\sum_{j=1}^{n} p_{j}x_{ij}$$

$$First Order Condition:$$

$$l_{i}=\frac{\alpha p_{i}z_{i}^{\alpha}l_{i}^{\alpha}\prod_{j\in\aleph_{i}} x_{ij}^{\left( 1-\alpha\right)a_{ij}}}{h} \Rightarrow l_{i}^{*}=\frac{\alpha p_{i}y_{i}}{h}$$

$$x_{ij}=\frac{\left( 1-\alpha\right){a_{ij}p}_{i}z_{i}^{\alpha}l_{i}^{\alpha}\prod_{j\in\aleph_{i}} x_{ij}^{\left( 1-\alpha\right)a_{ij}}}{p_{j}} \Rightarrow x_{ij}^{*}=\frac{(1-\alpha)a_{ij}p_{i}y_{i}}{p_{j}}$$

$substitute l_{i}^{*}andx_{ij}^{*} into y_{i}$:

$$y_{i}=z_{i}^{\alpha}\left( \frac{\alpha p_{i}y_{i}}{h} \right)^{\alpha}\left[ \prod_{j} \left( \left( 1-\alpha\right)\frac{a_{ij}p_{i}y_{i}}{p_{j}} \right)^{\left( 1-\alpha\right)a_{ij}} \right]$$

$$log-linearization: logz_{i}=\varphi_{i}$$

$$logy_{i}=\alpha\varphi_{i}+\alpha log\left( \alpha p_{i}y_{i} \right)-\alpha\log h+\left( 1-\alpha\right)a_{ij}log\left[ \left( 1-\alpha\right)\prod_{j} a_{ij}p_{i}y_{i} \right]-\left( 1-\alpha\right)a_{ij}log\prod_{j} p_{j}$$

$$logy_{i}=\alpha\varphi_{i}+\alpha log\alpha+\alpha logp_{i}+\alpha logy_{i}-\alpha logh+\left( 1-\alpha\right)a_{ij}log\left( 1-\alpha\right)+\left( 1-\alpha\right)\sum_{j} a_{ij}\log a_{ij}+\left( 1-\alpha\right)a_{ij}logp_{i}+\left( 1-\alpha\right)a_{ij}logy_{i}-\left( 1-\alpha\right)\sum_{j} a_{ij}\log\sum_{j} p_{j}$$

$$\left( 1-\alpha-(1-\alpha)a_{ij} \right)logy_{i} Constant returns to scale \alpha+(1-\alpha)a_{ij}=1$$

$$\left( 1-1 \right)logy_{i}=0$$

$$\alpha logh=\alpha\varphi_{i}+\left[ \alpha+\left( 1-\alpha\right)a_{ij} \right]logp_{i}-\left( 1-\alpha\right)a_{ij}logp_{j}+\alpha log\alpha+\left( 1-\alpha\right)log\left( 1-\alpha\right)+\left( 1-\alpha\right)\sum_{j} a_{ij}loga_{ij}$$

$$Define B=\alpha log\alpha+\left( 1-\alpha\right)log(1-\alpha)$$

$$\alpha logh=\alpha\varphi_{i}+logp_{i}-\left( 1-\alpha\right)a_{ij}logp_{j}+\left( 1-\alpha\right)\sum_{j} a_{ij}loga_{ij}+B$$

$$p_{i}=p_{j}$$

$$\alpha logh=\alpha\varphi_{i}+\left[ I-\left( 1-\alpha\right)A \right]logp_{i}+B+\left( 1-\alpha\right)\sum_{j} a_{ij}loga_{ij}$$

$$where A is input-output matrix$$

$$logh=\varphi_{i}+\left[ I-\left( 1-\alpha\right)A \right]\frac{logp_{i}}{\alpha}+\frac{B}{\alpha}+\frac{\left( 1-\alpha\right)}{\alpha}\sum_{j} a_{ij}loga_{ij}$$

$$influence vector V_{n}^{'}=\frac{\alpha}{n}\boldsymbol{1}^{'}\left[ I-\left( 1-\alpha\right)A \right]^{-1}$$

$$and V_{n}^{'}\boldsymbol{1}=1$$

$$premultiply by V_{n}^{'} and sum over i$$

$$V_{n}^{'}\boldsymbol{1}logh=V_{n}^{'}\varphi_{i}+\frac{1}{n}\sum_{i} logp_{i}+V_{n}^{'}\boldsymbol{1}\frac{B}{\alpha}+\frac{\left( 1-\alpha\right)}{\alpha}\sum_{i} \sum_{j} v_{i}a_{ij}loga_{ij}$$

$$h and B do not vary$$

$$logh=V_{n}^{'}\varphi_{i}+\frac{1}{n}\sum_{i} logp_{i}+\frac{B}{\alpha}+\frac{\left( 1-\alpha\right)}{\alpha}\sum_{i} \sum_{j} v_{i}a_{ij}loga_{ij}$$

$$define u\equiv\frac{1}{n}\sum_{i} logp_{i}+\frac{B}{\alpha}+\frac{\left( 1-\alpha\right)}{\alpha}\sum_{i} \sum_{j} v_{i}a_{ij}loga_{ij}$$

$$logh=V_{n}^{'}\varphi_{i}+u$$

$$define c=n\left( -\frac{B}{\alpha}-\frac{\left( 1-\alpha\right)}{\alpha}\sum_{i} \sum_{j} v_{i}a_{ij}loga_{ij} \right)$$

$$and \frac{1}{c}\left( p_{1}{,p}_{2},\ldots,p_{n} \right)^{\frac{1}{n}}=1$$

$${log}_{n}c=-\frac{B}{\alpha}-\frac{\left( 1-\alpha\right)}{\alpha}\sum_{i} \sum_{j} v_{i}a_{ij}loga_{ij}$$

$$\frac{1}{n}{log}_{n}\left( \prod_{i} p_{i} \right)={log}_{n}c , then$$

$$\frac{1}{n}\sum_{i} logp_{i}=-\frac{B}{\alpha}-\frac{\left( 1-\alpha\right)}{\alpha}\sum_{i} \sum_{j} v_{i}a_{ij}loga_{ij}$$

$$therefore u=0 and$$

$$\log h=V_{n}^{'}\varphi_{i} or y=V_{n}^{'}\varphi_{i}$$

 Q.E.D.

$$\mathbf{Proof of Derivative w.r.t input demands (Eq. 4):}$$

$$\frac{\partial x_{ij}}{\partial\alpha}=-\frac{\left( 1-\alpha\right)a_{ij}^{2}p_{i}\sum logx_{ij}\left( z_{i}l_{i} \right)^{\alpha}\prod_{j\in\aleph_{i}} x_{ij}^{\left( 1-\alpha\right)a_{ij}}}{p_{j}}-\frac{a_{ij}p_{i}\left( z_{i}l_{i} \right)^{\alpha}\prod_{j\in\aleph_{i}} x_{ij}^{\left( 1-\alpha\right)a_{ij}}}{p_{j}}+\frac{{(1-\alpha)a}_{ij}p_{i}log(z_{i}l_{i})\left( z_{i}l_{i} \right)^{\alpha}\prod_{j\in\aleph_{i}} x_{ij}^{\left( 1-\alpha\right)a_{ij}}}{p_{j}}$$

Since it is plausible that $\left( 1-\alpha\right)\log\left( z_{i}l_{i} \right)<\left( 1-\alpha\right)a_{ij}\sum_{j} logx_{ij}+1$, then:

$$\frac{\partial x_{ij}}{\partial\alpha}<0$$
